# Novelty in the Impact of Physical Therapy in a Known Case of Right-Sided Grade V Facial Nerve Palsy: A Case Report

**DOI:** 10.7759/cureus.30053

**Published:** 2022-10-08

**Authors:** Purva S Shahade, Purva H Mundada, Rakesh K Kovela, Pallavi Harjpal

**Affiliations:** 1 Physiotherapy, Ravi Nair Physiotherapy College, Datta Meghe Institute of Medical Sciences, Wardha, IND; 2 Physiotherapy, Nitte Institute of Physiotherapy, Nitte (Deemed to be University), Mangalore, IND

**Keywords:** recreational exercises, neuro-proprioceptive facilitation, rehabilitation, otitis externa, facial nerve palsy

## Abstract

Here we present a case study of a 75-year-old hypertensive elderly male suffering from right-sided malignant otitis externa with right-sided grade V facial nerve palsy. Malignant otitis externa is an uncommon but critically and extremely challenging kind of disease, along with the involvement of cranial nerves. The main aim of our case study is to highlight and explain the novelty impacts, efficacy, and role of physical therapy interventions and rehabilitation strategies in this case. The assessment consists of observations and structural impairments assessed through proper assessment strategies. The outcome measures used here are the House-Brackmann Facial Nerve Scale and the Sunnybrook Facial Grading Scale.

## Introduction

The facial nerve, which is our seventh cranial nerve, plays a very significant role in various multiple functional complexities such as mastication, communication as well as speech, etc which are a part of our day-to-day life [1,2]. The facial nerve carries motor, sensory, and parasympathetic functions, and it is prone to injury because of its long intraosseous course [3]. Facial nerve paralysis can be classified as either a central type or a peripheral type, based on the level of injury of the nerve. The central type leads to paralysis of the lower part of all facial muscles, mainly on the opposite side of the lesion. The upper facial muscles are mostly spared because of bilateral cortical connections. The peripheral type, which is the lower motor neuron lesion, usually produces whole facial paralysis on the exact same side of the lesion [4].

Facial palsy is a rare complication due to otitis externa, which is an infection of the external auditory canal. It is usually seen in patients having necrotizing otitis externa along with skull base osteomyelitis, mostly when there is a presence of comorbidities [5]. Unilateral facial nerve palsy is one of those depressing neurologic disorders that mimic a stroke [6]. Malignant otitis externa is an uncommon but critically and extremely challenging disease, along with the involvement of cranial nerves [7]. The case study presented here is of a 75-year-old hypertensive elderly male suffering from right-sided malignant otitis externa with right-sided grade V facial nerve palsy. Our case study aims to highlight and explain the novelty impacts, efficacy, and role of physical therapy interventions and rehabilitation strategies in this case. This case study revolves around the assessment, effectiveness, and novelty presented by physical therapy intervention strategies.

## Case presentation

Patient information

Here we presented a 75-year-old male patient who was apparently alright 15 days ago when he started complaining of right ear discharge, which was insidious in onset, progressive in nature, moderate in quantity, non-blood stained, non-foul-smelling and was followed by an episode of upper respiratory tract infection (URTI). The patient came to Acharya Vinoba Bhave Rural Hospital (AVBRH) with a chief complaint of left-sided generalized headache and earache, dull aching, throbbing type, radiating to the neck and occiput. He had right-sided facial weakness, which was insidious in onset, progressive in nature, and progressed to an inability to close his eyes and deviation of the angle of the mouth, which eventually resulted in an inability to chew food and drooling from the right side. The patient was unable to close his right eye for 15 days, and he has reduced hearing in his right ear. Following investigations, he was diagnosed with right-sided malignant otitis externa with right-sided facial nerve palsy.

Clinical findings

**Figure 1:**
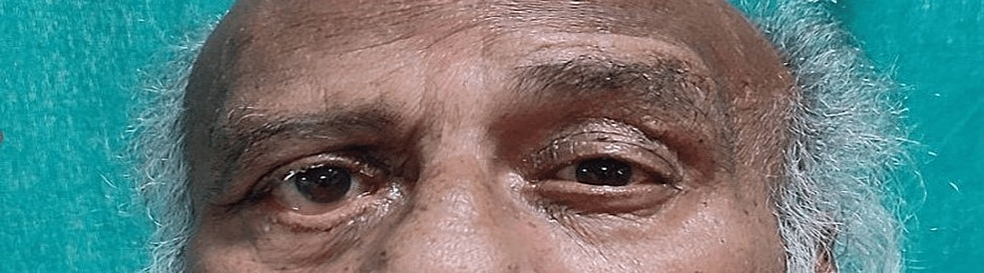
Raising eyebrows - unable to raise his right eyebrow

**Figure 2:**
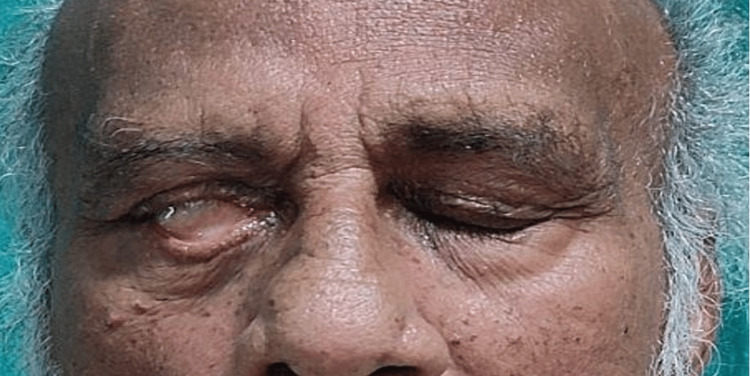
Closing of eyes - unable to close his right eye completely

**Figure 3:**
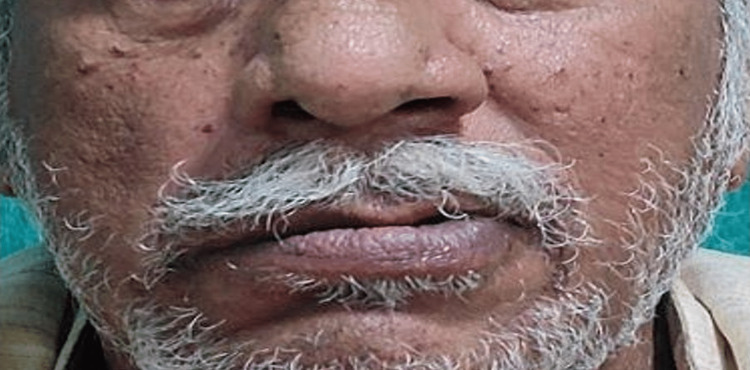
Corner of mouth dropped; deviation of the corner of the mouth towards left side - towards the non-paralyzed side of the face; unable to snarl; nasolabial crease absent

After obtaining consent, the patient was taken for examination. On observation, no abnormal findings were observable on the forehead. However, the patient was unable to raise his right eyebrow (Figure 1) and unable to close his eyes (Figure 2), with a deviation of the angle of the mouth, inability to chew food and drooling from the right side, nasolabial crease absent (Figure 3).

Physiotherapy management

The patient was discharged after two weeks, but for physical therapy treatment, he was instructed to come to the rehabilitation center for nine weeks, and then the home program was continued. Detailed physiotherapy management is given in Table 1.

**Table 1:** Week-wise physiotherapy management and home exercise program min: minutes, reps: repetitions, AAROM: active assisted range of motion

Intervention	Rationale	Intensity	Instructions	Week 1	Week 3	Week 5	Week 7	Week 9	Home exercise program
Soft Tissue Mobilisation - effleurage	For improving the circulation of facial muscles	Progress from 4 min to 5 min	Ask the patient to relax and sit comfortably	4 min twice a week	4 min thrice a week	5 min thrice a week	5 min - for 4 days per week	5 min - for 5 days per week	Not required
Facial expression exercises; for example- eye opening and closing	To re-educate the muscles as well as to enhance the circulation	Active Assisted	Focusing on the point which is at least 5 to 6 feet ahead of you on the ground while closing your eyes fully. These exercises should always be done along with the mirror biofeedback	5 reps per hour at least thrice a day	7 reps per hour at least thrice a day	9 reps per hour at least thrice a day	13 reps per hour at least thrice a day	15 reps per hour at least thrice a day	15 reps per hour at least thrice a day
AAROM - smile, eyebrow raise, scrunching face, frown, puckering of lips, (Figures 4-6)	To strengthen the muscles as well as to enhance the circulation	Isometric hold	These exercises should be done in front of the mirror to get visual biofeedback	10 reps thrice daily	13 reps thrice daily	15 reps thrice daily	17 reps thrice daily	20 reps thrice daily	20 reps thrice daily
Neuro-proprioceptive facilitation techniques for facial muscles - without resistance and with resistance	To activate and strengthen the facial muscles as much as possible	The therapist has to facilitate the muscle movement first and then provide resistance to the facial muscles as much as possible	This intervention started when the patient was able to activate his facial muscles independently at the end of the 2nd week	The patient was being taught about how he can activate his facial muscle independently	10 reps thrice daily	13 reps thrice daily	15 reps thrice daily	17 reps thrice daily	20 reps thrice daily
Electrical muscle stimulation	For re-educating the facial muscles	Interrupted galvanic to surged faradic	Keep calm and inform the therapist if any discomfort or burning sensation caused	30 reps - 2 sets- interrupted galvanic	40 reps - 2 sets- interrupted galvanic	40 reps - 2 sets- surged faradic	45 reps - 2 sets-surged faradic	50 reps - 2 sets- surged faradic	Not advisable as a home protocol
Recreational exercises- balloon blowing (Figure 7), drinking water with a straw, blowing bubbles with straw	To strengthen the facial muscles in a relaxing way through recreational activities	As per the comfort and willingness of the patient	Ask the patient to relax and enjoy these recreational activities as much as possible by creating a positive mindset.	5 reps thrice a day	10 reps thrice a day	13 reps thrice a day	15 reps thrice a day	17 reps thrice a day	20 reps thrice a day

**Figure 4:**
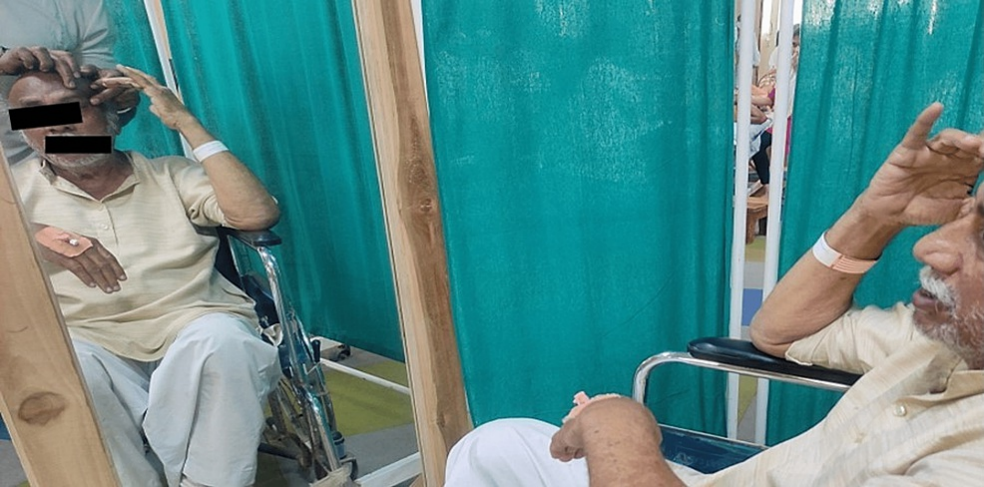
Facial exercises - assisted raising of eyebrows with mirror biofeedback

**Figure 5:**
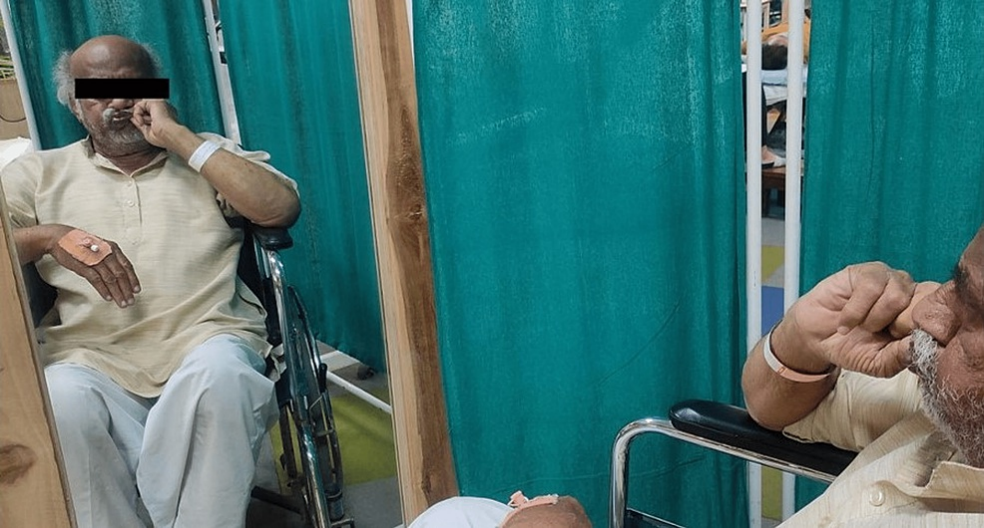
Facial exercise - assisted closure of the mouth

**Figure 6:**
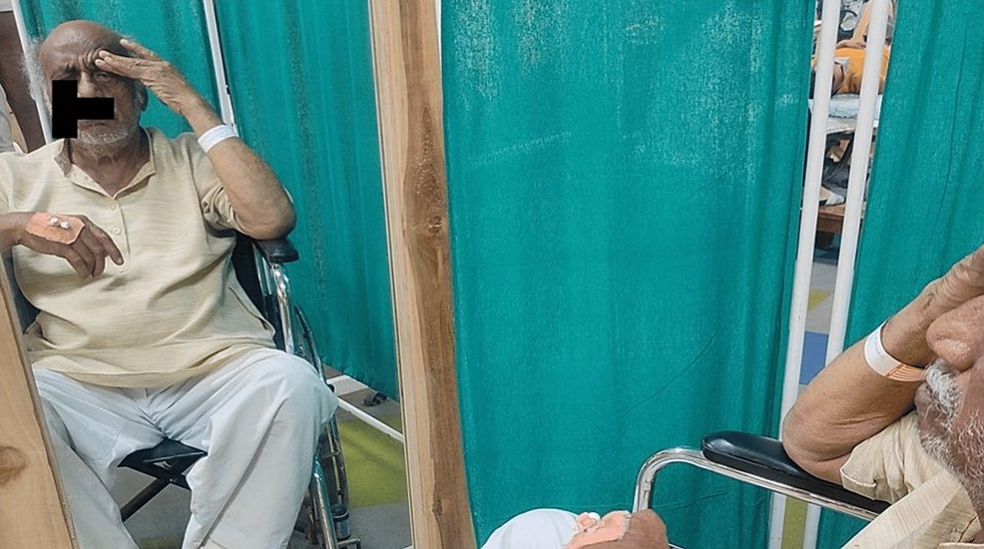
Facial exercises - assisted frowning of eyebrows with mirror biofeedback

**Figure 7:**
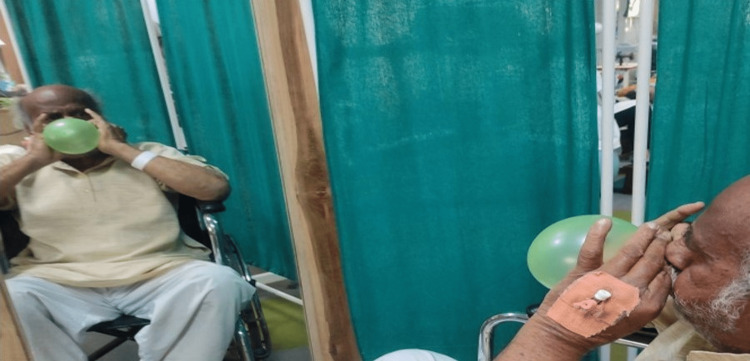
Blowing a balloon

Outcome measures

The Sunnybrook grading scale is a type of clinical grading system that is used for facial function in patients with facial palsy. This scale includes 13 items, among which three are resting, five are related to voluntary movement, and five are about synkinesis movement. The total score ranges from 0-100, among which the score range for resting symmetry is 0-20, the score range for voluntary symmetry is 20-100, and synkinesis is 0-15 [8,9].

**Figure 8:**
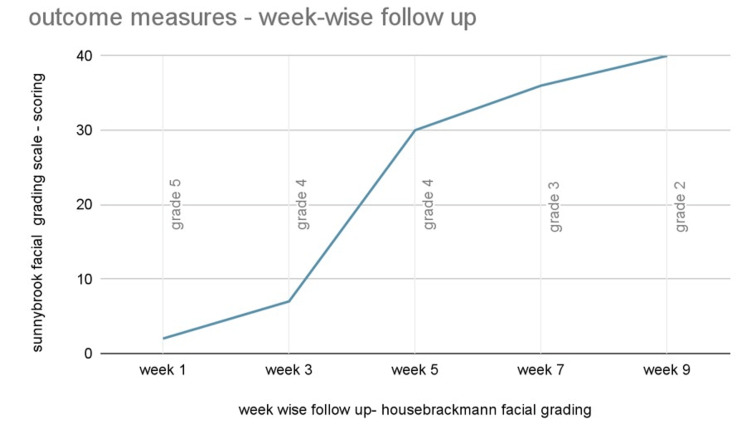
Week-wise follow-up in terms of Sunnybrook grading scale and House-Brackmann grading system X-axis denotes scoring of House Brackmann scale - week wise Y-axis denotes the grading of the Sunnybrook grading scale - week wise

The House-Brackmann grading system is one of the most widely used tools or scales. It is considered the universal standard all around the world. This system assesses four regions of the face and assigns a score or grading from 1 to 6 for the degree and extent of movement. A scale of 0 to 3 is scored for the synkinesis problems, and the addition of these two types of scores resulted in a final score of 4 to 24, which was afterward converted to grading [10,11]. Week-wise follow-up in terms of Sunnybrook grading scale and House-Brackmann grading system is given in Figure 8.

## Discussion

The comparative study presented by Dabiri et al. was about the paralysis of the facial nerve in the disease malignant otitis externa. In this study, they compared the clinical and paraclinical findings. They concluded in their study that the facial nerve canal erosion along with the nasopharyngeal extension of the inflammation may be significant in being the predictors of the dysfunction of the facial nerve. They also mentioned in their study that the elevation in the erythrocyte sedimentation rate is related to the enhanced risk of paralysis of the facial nerve and that aggressiveness in the medical management protocol is mandatory and crucial [7].

A case report presented by Aliyya Badaruddin and May May Choo on facial nerve palsy in a patient with otitis externa concluded that facial palsy in patients with otitis externa is a usually uncommon complication and can easily be prevented with proper medical management. Proper medical management consists of recognition of the disease at an early stage, referrals, treatments along with optimization of the comorbidities present, and they also stated that necrotizing otitis externa should be suspected in otitis externa patients who have diabetes, who belong to a geriatric population, and do not respond to the orally given antibiotic treatment [5].

Somnath Saha et al. presented a unique case of malignant otitis externa involving cranial nerves bilaterally. They concluded in their study that during the advanced stage of the disease, the base of the skull may be involved, usually with multiple lower cranial nerve palsies. In their case report, there was the involvement of the contralateral sixth along with the twelfth nerve. The most probable cause of this was the involvement of contralateral nerves by skip lesions of the microabscesses at the base of the skull. However, they also mentioned that this data is hypothetical and needs more research [12].

The present case study revolves around the assessment, effectiveness, and novelty presented by physical therapy intervention strategies. This case study includes the assessment and intervention strategies that were used. The assessment consists of observations and structural impairments assessed through proper assessment strategies. The outcome measures used here are the House-Brackmann Facial Nerve Scale and the Sunnybrook facial grading scale. The therapeutic interventions include medications and novelty, which are incorporated into classical physiotherapy management. The physical therapy management consists of a week-wise systematically designed protocol, which consists of soft tissue mobilization-effleurage, and facial expression exercises, for example, eye-opening and closing, active assisted range of motion (AAROM)-smile, eyebrow raise, scrunching face, frown, puckering of lips, neuro-proprioceptive facilitation techniques for facial muscles, electrical muscle stimulation, and recreational exercises - balloon blowing, drinking water with a straw, and blowing bubbles with a straw.

## Conclusions

This is a classic case of right-sided malignant otitis externa with a right-sided grade V facial nerve palsy. Though it is a depressing condition for the patient and the family members, it can be cured indeed by designing proper physiotherapy management, which includes novelty in interventions and recreational activities were added too, due to which the quality of life can be enhanced. The physical therapy management consists of a week-wise systematically designed protocol, which consists of soft tissue mobilization-effleurage, and facial expression exercises, for example, eye-opening and closing, AAROM-smile, eyebrow raise, scrunching face, frown, puckering of lips, neuro-proprioceptive facilitation techniques for facial muscles, electrical muscle stimulation, and recreational exercises - balloon blowing, drinking water with a straw, blowing bubbles with a straw.
